# Rats with Chronic, Stable Pulmonary Hypertension Tolerate Low Dose Sevoflurane Inhalation as Well as Normal Rats Do

**DOI:** 10.1371/journal.pone.0154154

**Published:** 2016-05-04

**Authors:** Xiaoqing Yin, Lu Wang, Gang Qin, Hui Luo, Xiao Liu, Fan Zhang, Zhi Ye, Junjie Zhang, E. Wang

**Affiliations:** Department of Anesthesiology, Xiangya Hospital, Central South University, Changsha, China; Vanderbilt University Medical Center, UNITED STATES

## Abstract

**Background:**

The effects of low concentration of sevoflurane on right ventricular (RV) function and intracellular calcium in the setting of pulmonary arterial hypertension (PAH) have not been investigated clearly. We aim to study these effects and associated signaling pathways in rats with PAH.

**Methods:**

Hemodynamics were assessed with or without sevoflurane inhalation in established PAH rats. We analysis the classic RV function parameters and RV-PA coupling efficiency using steady-state PV loop recordings. The protein levels of SERCA2, PLB and p-PLB expression was analyzed by western blot to assess their relevance in PAH.

**Results:**

Rats with PAH presented with RV hypertrophy and increased pulmonary arterial pressure. The values of Ea, R/L ratio, ESP, SW, PRSW, +dP/dt_max_ and the slope of the dP/dtmax-EDV relationship increased significantly in PAH rats (*P*<0.05). Sevoflurane induced a concentration-dependent decrease of systemic and pulmonary blood pressure, HR, RV contractility, and increased the R/L ratio in both groups. Sevoflurane reduced the expression of SERCA2 and increased the expression of PLB in both groups. Interestingly, sevoflurane only reduced the p-PLB/PLB ratio in PAH rats, not in normal rats.

**Conclusions:**

Rats with chronic, stable pulmonary hypertension tolerate low concentrations of sevoflurane inhalation as well as normal rats do. It may be related to the modulation of the SERCA2-PLB signaling pathway.

## Introduction

Pulmonary arterial hypertension (PAH) is characterized by chronically increased intravascular pressure and resistance, which results in right ventricular (RV) hypertrophy, dilatation, right heart failure, and ultimately death[1–3]. PAH is a major risk factor for mortality in patients undergoing surgery. In cardiac surgery, PAH patients exhibit higher mortality than patients without PAH[4, 5]. In non-cardiac surgery, PAH is associated with worse perioperative outcomes. In-hospital mortality of severe PAH patients is up to 10%. [6, 7] The choice of anesthetic agent is crucial in the perioperative management of patients with PAH. Midazolam, fentanyl, neuromuscular blocking agents with minimal hemodynamic effects and a small dose of propofol has been recommended for induction of anesthesia in pediatric PAH patients[8, 9]. Sevoflurane is a widely used volatile agent that affords cardioprotection and neuroprotection[10,11]. However, sevoflurane can produce dose-dependent decreases of both pulmonary and systemic pressures.[12, 13]. Little is known about the effects of sevoflurane on RV function in patients with PAH.

The monocrotaline (MCT)-induced PAH in rats is the most commonly used animal model. A single injection of MCT can result in injury of the vascular endothelium, pulmonary hypertension, RV hypertrophy and failure within 3 to 4 weeks [14–16].

Growing evidence implicates that downregulation of sarcoplasmic reticulum Ca^2+^-ATPase 2a (SERCA2a) plays an important role in PAH. Alterations in Ca^2+^ homeostasis not only stimulate pulmonary vascular proliferation but also impact RV myocardial contractility [14, 17]. The activation of SERCA2a depends on the phosphorylation of phospholamban (PLB) at Ser16 and at Thr17, allowing for increased Ca^2+^ pumping into the sarcoplasmic reticulum[18]. It is unclear whether the SERCA2a–PLB pathway is important to RV function in PAH, especially with sevoflurane administration.

We hypothesized that sevoflurane inhalation would affect RV function and the SERCA2-PLB pathway is affected in MCT induced PAH model. Primary end points are RV end-systolic elastance (Ees), preload recruitable stroke work (PRSW), and the slope of the relation of the maximum derivative of RV pressure (dP/dt_max_) to end-diastolic volume (EDV), effective arterial elastance (Ea), and RV-PA coupling efficiency assessed by Ees/Ea ratio. [19] SERCA2, PLB, p-PLB levels are compared after sevoflurane inhalation between normal and PAH animals.

## Methods

### Animals

All experiments received prior approval by the Ethics Committee for Animal Research of Xiangya Hospital (201303311). All surgery was performed under proper anesthesia, and all efforts were made to minimize suffering. Adult male Sprague-Dawley rats (SPF grade, Xiangya Medical College, Changsha, HN, China) weighing between 250 and 280 g were fed with the same bacteria-free diet and water and housed at 23°C on a 12 h/12 h dark/light cycle.

### Modeling and grouping

Rats were treated with solvent of 1% monocrotaline (Crotaline C2401, Sigma-Aldrich, Buchs, Switzerland) by a single intraperitoneal injection at a dose of 60 mg/kg administered 28 days before the experiment. Schematic diagram depicting the experimental protocols used in the sevoflurane inhalation experiments was shown in S1 Fig. Monocrotaline was dissolved in ethanol and diluted with normal saline. After injection of MCT or NS, the rats received high-quality care by laboratory animal technicians. The professional staff and members of our research team inspected the animals every day and disposed in time. Occasionally the rats were very sick and stopped eating before 28^th^ day, then they were given early euthanasia. If the rat was severely sick and stopped eating, the protocol of the early euthanasia was taken. Cervical dislocation was done under ketamine anesthesia. The rats with or without monocrotaline injection were divided randomly into groups with or without sevoflurane inhalation before instrumentation. Therefore, rats were divided into 4 groups: group NS (n = 8), group MCT (n = 8), group NS-sevo (n = 12), and group MCT-sevo (n = 13). All observers/recorders were blinded to animal assignments.

### Surgical preparation

The rats were anesthetized with ketamine (60 mg/kg) by intraperitoneal injection and placed on an electrical heating pad to maintain body temperature at 37°C to 38°C. Then, the rats were intubated by tracheotomy with a 16-gauge catheter. The left femoral artery and the right femoral vein were catheterized with 24-gauge catheters to record systemic arterial pressure and to infuse lactated Ringer’s solution at 10 ml·kg^-1^·h^-1^ throughout the experiment. Before opening the chest, 1 mcg/kg sufentanil (Renfu, Yichang, Hubei, CHN) were given intravenously and then infuse sufentanil 1 mcg kg-1•h-1 to ensure the maintenance of anesthesia. The rats were mechanically ventilated (Model 683, Harvard Apparatus, USA) initially with 100% oxygen at a tidal volume of 5 ml/kg and a respiratory rate of 70–80 min^-1^. A positive end-expiratory pressure of 3 cmH_2_O was used to prevent atelectasis. After midline sternotomy, the pulmonary artery and right ventricle were catheterized and parameters measured.

### Experimental protocol

A PE catheter was inserted into the pulmonary artery to monitor pulmonary arterial pressure. Then, the right ventricle was catheterized with a 1.9 F conductance pressure-volume (PV) catheter (Millar Instruments Inc., USA) along the long axis. The correct position was determined by online visualization of the PV loops. Right ventricular function was determined by PV loops and was continuously monitored throughout the experimental procedure with a PowerLab system (ADInstruments, Australia) via a conductance PV catheter placed in the RV. During the procedure, right ventricular pressure and volume as well as steady-state and dynamic PV loops during occlusion of the IVC were continuously recorded. For each condition, data were obtained after a 5-second period while mechanical ventilation was suspended, followed by a transient reduction of RV preload through IVC occlusion (<5 sec), and approximately 10–20 cardiac cycles were selected and used for later analysis with LabChart analysis software (ADInstruments, Australia).

After 30 min of postoperative stabilization, rats in groups with sevoflurane inhalation were exposed to stepwise increasing doses (0.5%, 1.0%, and 1.5%) of sevoflurane, each dose for periods of 30 min (corresponding timing T1, T2, T3). We chose this dose because lower dose of sevoflurane is usually used for critical patients with PAH to avoid systemic hypotension in clinical settings [20]. Rats in the other groups received oxygen without sevoflurane and were maintained for the same times. Hemodynamic data and cardiac function parameters were collected before sevoflurane inhalation (baseline T0) and at each concentration after 30 min of equilibration. After animals were sacrificed by exsanguination, the organs were harvested for analysis.

### Measurements of hemodynamic and RV cardiac function

Data were continuously recorded and stored for later analysis. For each baseline and experimental condition, at least 20 consecutive cardiac cycles were selected and used for analysis. From the systemic and pulmonary arterial pressure recordings, we derived standard hemodynamic variables: HR, systolic blood pressure (SBP), diastolic blood pressure (DBP), mean systemic arterial pressure (MAP), systolic pulmonary arterial pressure (sPAP), diastolic pulmonary arterial pressure (dPAP), R/L ratio.

From steady-state PV loop recordings, we derived the following classic RV function parameters: RV end-systolic pressure (ESP), RV end-diastolic pressure (EDP), RV end-systolic volume (ESV), RV end-diastolic volume (EDV), RV stroke volume (SV), RV cardiac output (CO), RV ejection fraction (EF), RV stroke work (SW), and Ea., assuming that the left ventricular CO equals the RV CO. RV contractility was quantified by three parameters: Ees (the slope of end-systolic pressure-volume relations), PRSW, and the slope of the dP/dt_max_-EDV relation. These parameters are useful to evaluate RV contractile performance because they are load independent. Finally, RV-PA coupling efficiency was assessed using Ees/Ea.

### Morphometric analysis

After sacrifice of the rats, the hearts were removed and frozen for 15 min at -20°C and then cut into transverse slices of 2 mm and photographed. The midventricular slice was used to estimate the ratio of right ventricular over left ventricular wall thickness using an image analysis program. The weight of RV free wall and left ventricle (LV) plus septum (S) were determined by reassembling the respective wall slices, and the weight ratio of RV to (LV+S) was used to estimate the degree of RV hypertrophy. The hearts and left lungs were removed and fixed in 4% paraformaldehyde and embedded in paraffin for histological analysis. The paraffin-embedded sections were processed for hematoxylin and eosin staining.

### Western blot analysis

The samples of right ventricle were homogenized in RIPA Lysis Buffer containing 20 mM Tris–HCl (pH 7.5), 150 mM NaCl, 1 mM Na2EDTA, 1 mM EGTA, 1% Triton, 2.5 mM sodium pyrophosphate, 1 mM beta-glycerophosphate, 1 mM Na3VO4, 1 μg/ml leupeptin (20 ml/g tissue) and 1 mM phenylmethylsulfonyl fluoride (Wellbiology, China) containing PMSF(1Mm) and placed on ice for 30 min. The homogenates were centrifuged at 14,000 g for 30 min at 4°C to remove cell debris, and protein concentrations were measured with a BCA protein assay kit (Wellbiology, China). Proteins (20μg) were resolved by 12% sodium dodecyl sulfate–polyacrylamide gel electrophoresis (SDS-PAGE) and then transferred to polyvinylidene difluoride (PVDF) membranes. After blocking with 5% non-fat milk in Tris-buffered saline-0.1% Tween 20 (TBST), membranes were immunoblotted overnight at 4°C with primary antibodies against SERCA2, PLB, p-PLB and Na-K-ATPase (1:1000; Cell Signaling Technology, USA), followed by incubation with the corresponding secondary HRP-conjugated antibody (1:4000; ComWin Biotech, China) at room temperature for 60 min. The immunoreactivity was detected by ECL (Thermo Scientific Pierce, USA) and exposed to X-ray film. The signal intensity was normalized to Na-K-ATPase expression.

### Statistical analysis

Statistical analysis was performed using GraphPad Prism 6 software (GraphPad, La Jolla, CA, USA). The distributions of the continuous variables were expressed as means ± SD. Morphometric data, baseline hemodynamic data between control and monocrotaline group were compared using a two-sample t-test. For hemodynamic and RV function data, repeated-measures ANOVA was used to evaluate difference over time. Comparisons were intra-group and between group, followed by post hoc LSD tests for multiple comparisons. Significance was assumed when the *P* value was less than 0.05.

## Results

### Morphometric analysis

**Fig 1:**
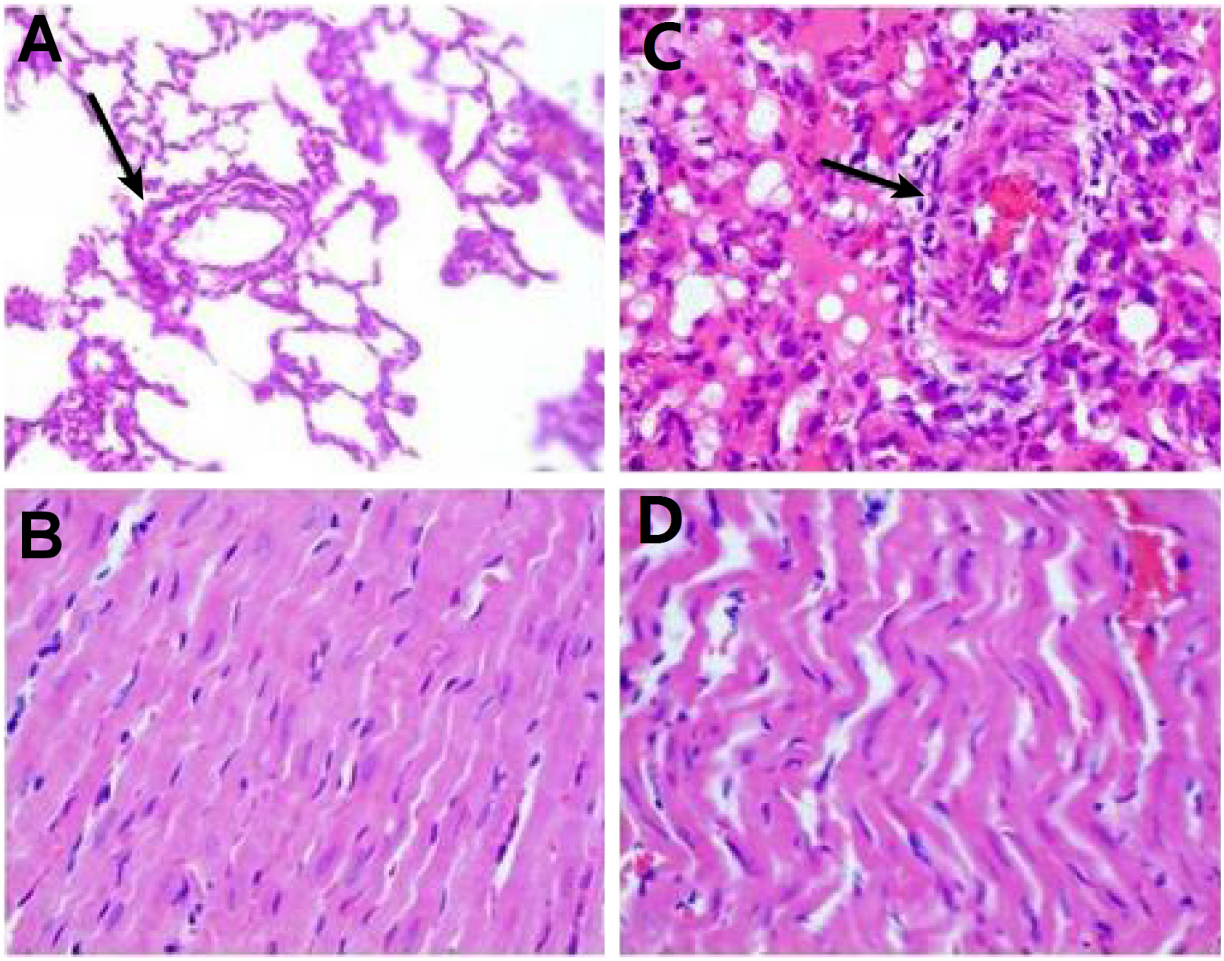
HE staining of lung (A) and right ventricle (C) from a normal rat and lung (B) and right ventricle (D) from a PAH rat. The black arrows show the pulmonary arterioles. PAH rats exhibited markedly thickened pulmonary arterioles, narrowed lumen, and hypertrophic endothelial cells, with a dense inflammatory infiltration around the arterioles. The RV myocardial cells of PAH rats were hypertrophic and exhibited a disorganized arrangement with different sizes of nuclei.

The number of the rats who became very ill is less than 5% of total animals. There were two animals died prior to the experiment endpoints. The reason might be over dosage of anesthetics. Midventricular transverse sections of hearts from a normal rat (A) and a PAH rat (B) were shown in S2 Fig. RV wall thickness increased in the PAH rat. The body weights (BW) of rats at 28 days after MCT injection were significantly lower than those of control rats. The heart weight (HW) and RV weight of MCT-injected animals were significantly higher than those of control rats, whereas LV and septal (LV+S) weight was unaffected by MCT treatment. The RV to (LV+S) weight ratio increased significantly in MCT rats. The ratio of the right over left ventricle wall thickness was significantly different between normal rats and PAH rats, as was the ratio of heart weight over body weight. (Table 1) RV wall thickness increased significantly in MCT-treated rats and MCT-injected rats had markedly thickened pulmonary arterioles, narrowed lumens, hypertrophic endothelial cells, and a dense inflammatory infiltration around the arterioles. RV myocardial cells were hypertrophic and of inordinate arrangement after MCT treatment. (Fig 1).

**Table 1:** Cardiac morphometric data *(n = 12)*.

*parameters*	*Normal saline*	*Monocrotaline*	*P value*
BW, g	376.67 ± 27.41	316.75 ± 17.43	0.000
HW, mg	896.7 ± 76.0	1045.7 ± 150.9	0.007
RV weight, mg	183.0 ± 21.1	351.9 ± 79.2	0.000
LV+S weight, mg	671.1 ± 60.3	653.7 ± 87.6	0.577
HW/BW, mg/g	0.24 ± 0.01	0.33 ± 0.04	0.000
RV/(LV+S) weight	0.27 ± 0.02	0.54 ± 0.10	0.000
RV/LV wall thickness	0.37 ± 0.03	0.55 ± 0.04	0.000

Values are expressed as means ± SD; n, number of rats; BW, body weight; HW, heart weight; RV, right ventricle; LV, left ventricle; S, septum. *P* values from Student’s unpaired *t*-test.

### Measurements of hemodynamic and RV cardiac function

**Fig 2:**
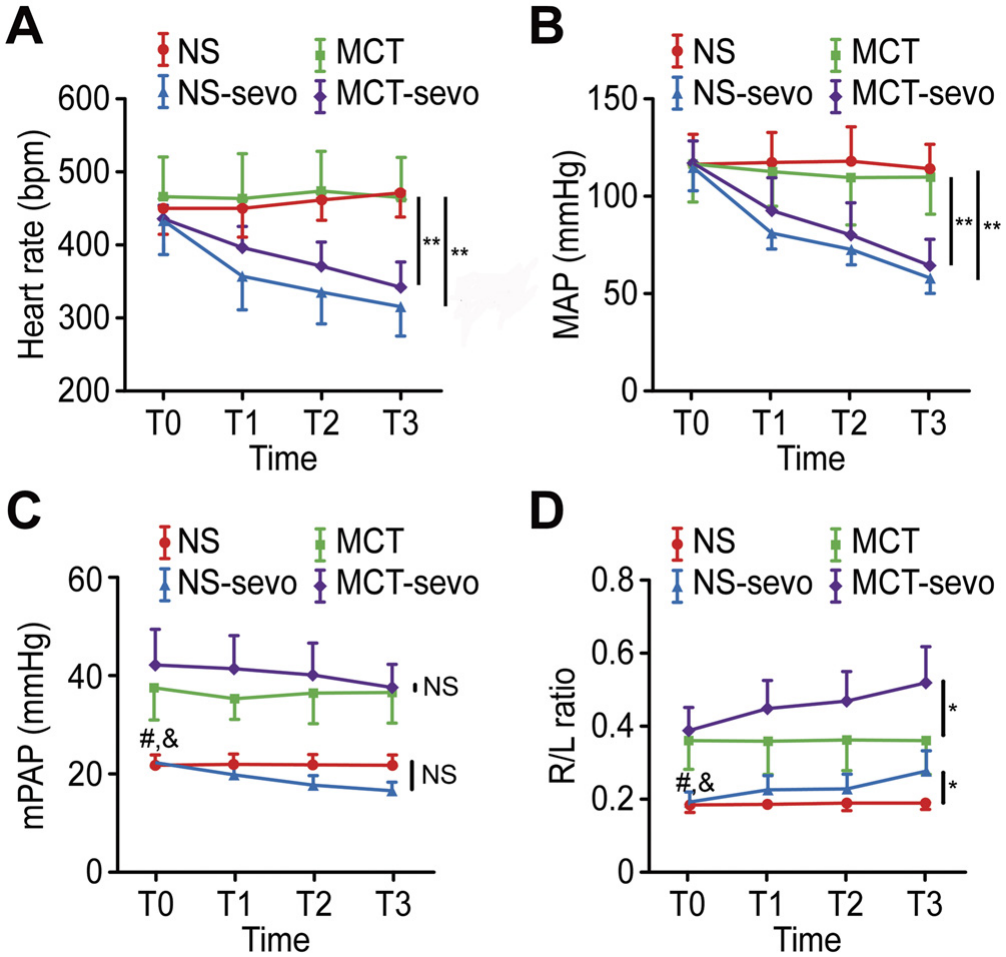
Values are expressed as means±SD. *P* values from repeated measures ANOVA; *, group NS compared with group NS-sevo, group MCT compared with MCT-sevo, *P<*0.05,**, group NS compared with group NS-sevo, group MCT compared with MCT-sevo, *P*<0.01, NS, group NS compared with group NS-sevo, group MCT compared with MCT-sevo, no significant. #, group NS compared with group MCT at T0, *P*<0.05, &, group NS-sevo compared with MCT-sevo at T0, *P*<0.05. HR, heat rate; MAP, mean systemic arterial pressure; mPAP, mean pulmonary arterial pressure; R/L ratio, ratio of systolic pulmonary arterial pressure over systolic blood pressure.

In normal saline and MCT treated rats, HR and MAP decreased significantly and gradually in a concentration-dependent manner with sevoflurane administration (*P*<0.01). In MCT and MCT-sevo groups, mean pulmonary artery pressure significantly increased at T0 (*P*<0.05), whereas the R/L ratio of ventricular pressure was significantly higher than those of NS group and NS-sevo group at T0 (*P*<0.05). But mPAP didn’t change significantly after sevoflurane inhalation in any group. The R/L ratio of ventrical pressure increased significantly with sevoflurane administration in both of normal and PAH rats (*P*<0.05). (Fig 2)

**Fig 3:**
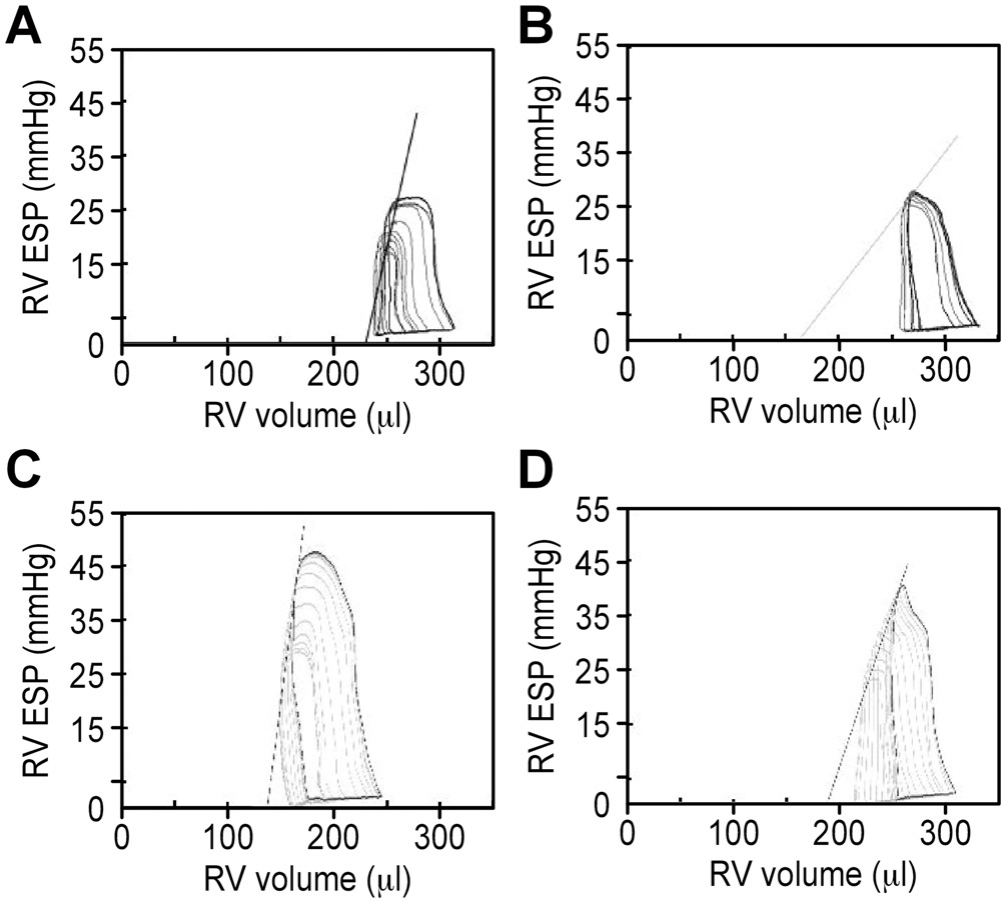
Right ventricle (RV) pressure-volume (PV) loops. Representative recordings of RV PV loops in a normal rat (A) and a PAH rat (B) during baseline condition and n a normal rat (C) and a PAH rat (D) 1.5% sevoflurane inhalation. Solid lines (black arrows) represent end-systolic pressure volume-relationship (ESPVR). RV ESP, right ventricle end-systolic pressure.

At baseline (T0), MCT-sevo groups exhibited significantly increased ESP, Ea, R/L ratio, SW, PRSW, +dP/dtmax and the slope of the dP/dtmax -EDV relationship, slightly increased EDP, and slightly decreased CO, SV, and EF compared to normal rats. 1.5% sevoflurane administration did not change Ea, but the Ees/Ea ratio, Ees, PRSW, and dP/dt_max_-EDV was decreased in both NS-sevo and MCT-sevo groups. Sevoflurane resulted in a decreased ejection capacity with reduced SV, CO, SW and EF, associated with increased ESV and EDV. But the values of ESP, SW, Ees, PRSW, and dP/dt_max_-EDV, and Ea MCT-sevo group remained significantly higher than those in NS-sevo group after 1.5% sevoflurane inhalation. (Table 2). RV PV loop demonstrated that sevoflurane reduced area, shifted loops rightward, decreased ESP, and decreased Ees in both normal and PAH rats (Fig 3).

**Table 2:** The parameters of right ventricular function.

Parameter	Group	T0	T1	T2	T3
**ESP**	NS-sevo	31 ± 3	28 ± 4^a^	25 ± 2^a^^b^	24 ± 2^a^^b^^c^
**(mmHg)**	MCT-seco	49 ± 7^d^	45 ± 8^a^	43 ± 9^a^^b^	41 ± 10^a^^b^
**EDP**	NS-sevo	2.24 ± 0.83	2.23 ± 1.09	2.08 ± 0.95	1.98 ± 0.99
**(mmHg)**	MCT-seco	3.03 ± 1.84	3.13 ± 1.48	2.40 ± 0.79	2.48 ± 0.94
**ESV**	NS-sevo	207±50	244±49 ^a^	272±56 ^a^^b^	298±58 ^a^^b^^c^
**(ul)**	MCT-sevo	222±43	257±61 ^a^	287±62 ^a^^b^	307±70 ^a^^b^^c^
**EDV**	NS-sevo	251±56	290±61 ^a^	307±61 ^a^^b^	334±64 ^a^^b^^c^
**(ul)**	MCT-sevo	279±49	313±70 ^a^	333±70 ^a^^b^	351±71 ^a^^b^^c^
**SV**	NS-sevo	64 ± 17	66 ± 20	58 ± 16 ^a^^b^	52 ± 14 ^a^^b^^c^
**(ul)**	MCT-sevo	68 ± 16	70 ± 16	65 ± 16^a^^b^	59 ± 18^a^^b^^c^
**CO**	NS-sevo	27.63 ± 8.56	23.68 ± 6.85^a^	19.25 ± 4.87^a^^b^	16.33 ± 4.36^a^^b^^c^
**(ml/min)**	MCT-sevo	28.56 ± 6.38	26.32 ± 5.17^a^	22.95 ± 5.16^a^^b^	19.80 ± 5.84^a^^b^^c^
**EF**	NS-sevo	27.23 ± 4.99	23.96 ± 5.10^a^	19.43 ± 3.30^a^^b^	16.02 ± 2.30^a^^b^^c^
**(%)**	MCT-sevo	26.27 ± 4.36	24.23 ± 4.20^a^	20.75 ± 4.39^a^^b^	17.88 ± 5.03^a^^b^^c^
**SW**	NS-sevo	1574±531	1457±751	1091±442 ^a^^b^	848±325 ^a^^b^^c^
**(mmHg٭ul)**	MCT-sevo	2249±685 ^d^	2219±653	1784±751 ^a^^b^	1616±744 ^a^^b^^c^
**Ea**	NS-sevo	0.50 ± 0.14	0.45 ± 0.13	0.45 ± 0.12	0.46 ± 0.11
**(mmHg/ul)**	MCT-sevo	0.79 ± 0.30^d^	0.71 ± 0.29^a^	0.72 ± 0.29^a^	0.71 ± 0.27^a^
**Dp/dt max**	NS-sevo	2619.6 ± 702.9	1584.4 ± 329.4 ^a^	1264.9 ± 147.5 ^a^^b^	1034.0 ± 110.5 ^a^^b^^c^
**(mmHg/s)**	MCT-sevo	3359.5 ± 491.1^d^	2572.6 ± 399.1 ^a^	2194.2 ± 407.9 ^a^^b^	2080.8 ± 366.8 ^a^^b^^c^
**Ees**	NS-sevo	0.67±0.12	0.57±0.13 ^a^	0.50±0.09 ^a^^b^	0.48±0.05 ^a^^b^^c^
**(mmHg/ul)**	MCT-sevo	0.95±0.42 ^d^	0.75±0.39 ^a^	0.64±0.18 ^a^^b^	0.58±0.16 ^a^^b^^c^
**PRSW**	NS-sevo	23.7±2.5	20.5±1.3 ^a^	18.1±0.02 ^a^^b^	17.1±0.03 ^a^^b^^c^
**(mmHg)**	MCT-sevo	31.5±4.9 ^d^	25.3±6.1 ^a^	21.8±5.3 ^a^^b^	19.4±5.5 ^a^^b^^c^
**Dp/dt max-EDV**	NS-sevo	21.1±1.8	18.8±0.7 ^a^	17.1±1.0 ^a^^b^	15.3±0.3 ^a^^b^^c^
**(mmHg/s/ul)**	MCT-sevo	41.3±9.3 ^d^	37.9±7.6 ^a^	33.7±8.2 ^a^^b^	30.3±7.0 ^a^^b^^c^
**Ees/Ea**	NS-sevo	1.44±0.08	1.30±0.03 ^a^	1.17±0.1 ^a^^b^	1.09±0.01 ^a^^b^^c^
	MCT-sevo	1.42±0.33	1.23±0.37 ^a^	1.11±0.35 ^a^^b^	1.02±0.23 ^a^^b^^c^

Values are expressed as means±SD. group NS-sevo, n = 12; group MCT-sevo, n = 13. ESP, end-systolic pressure; EDP, end-diastolic pressure; ESV, end-systolic volume; EDV, end-diastolic volume; dP/dt max, maximum dP/dt; Ea, effective arterial elastance; SV, stroke volume; CO, cardiac output; EF, ejection fraction; SW, stroke work; Ees, end-systolic elastance; PRSW, preload recruitable stroke work; dP/dtmax-EDV, slope of the maximum derivative of pressure (dP/dtmax)—end-diastolic volume (EDV) relationship; Ees/Ea, RV-pulmonary arterial (PA) coupling. P values from repeated-measures ANOVA.

^a^ compared to T0, *P*<0.05;

^b^ compared to T1, *P*<0.05;

^c^ compared to T2, *P*<0.05;

^d^ compared with the normal-sevo group at T0, *P*<0.05 from Student's t test.

#### Western blot analysis

**Fig 4:**
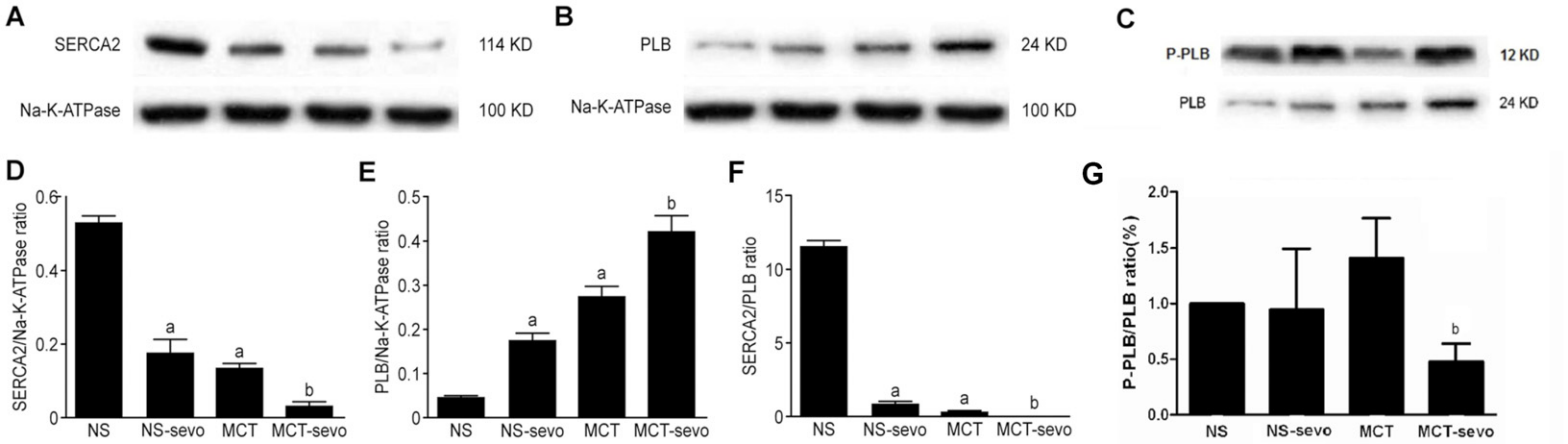
The effects of sevoflurane on the expression of SERCA2 (A,D), PLB (B, E), SERCA2/PLB ratio (F) p-PLB(C) and p-PLB/PLB ratio(G) in the right ventricle. The results are expressed as the mean±SD, n = 3. ^a^*P<*0.05 vs. NS; ^b^*P<*0.05 vs. MCT.

Levels of SERCA2 and PLB were examined by Western blotting. Compared with the control group, the levels of SERCA2 were reduced, whereas PLB was increased in the MCT group (*P*<0.001). The combined changes of the two proteins resulted in a decrease in SERCA2/PLB ratio in PAH rats (*P*<0.05). After sevoflurane inhalation, the protein expression of SERCA2 was reduced, whereas that of PLB increased, resulting in a decreased SERCA2/PLB in all rats treated with sevoflurane (all *P*<0.05). Interestingly, sevoflurane only diminished p-PLB/PLB ratio in PAH rats, not in normal rats (*P*<0.05). (Fig 4)

## Discussion

Our study was designed to examine the pulmonary hemodynamics and right ventricular function when low concentration of sevoflurane is administered to rats with or without pulmonary hypertension. We also investigated whether SERCA2-PLB signaling pathway is involved in this process. First, the pulmonary and systemic hemodynamic were similarly influenced by sevoflurane in rats with and without pulmonary hypertension. Second, sevoflurance affects RV contractility, and RV-PA coupling efficiency similiarly in both normal and PAH rats. Finally, the SERCA2-PLB signaling pathway might play a role in PAH pathogenesis and in the effects of sevoflurane on PAH or non-PAH rats.

Previous studies in MCT-treated animals have shown a progressive development of PAH and RV hypertrophy consistent with our results.[21, 22] Sevoflurane produced a similar concentration-dependent depression of systemic hemodynamics in normal and PAH rats. There were no significant differences in baseline systemic hemodynamic between the normal rats and those with PAH. Heart rate and mean arterial pressure decreased similarly in the two types of rats during inhalation of sevoflurane from 0.5% to 1.5%. Previous studies showed that sevoflurane can produce a dose-dependent decrease in pulmonary and systemic hemodynamics and depression of RV function at doses above 1.0 minimum alveolar concentration (MAC)[13, 14]. In our study, the mean pulmonary artery pressure decreased and the R/L ratio of ventricular pressure increased after sevoflurane inhalation in both normal and PAH rats. These findings indicate that the pulmonary vascular tone of PAH is not more sensitive to sevoflurane than that of the normal pulmonary circulation.

RV function in both clinical and experimental studies is commonly assessed by RV EF or dP/dt_max_, but both parameters are highly dependent upon preload and afterload in addition to contractility[23, 24]. RV contractility is better evaluated by Ees, PRSW, and dP/dt_max_-EDV in PV loops analysis because the three variables are load independent[23, 25]. In the current study, MCT-treated rats exhibited intact RV contractility and RV CO and EF were maintained. Previous studies have shown that the right ventricle can adapt to PAH by an increase in contractility (homeometric autoregulation) and maintain CO[14, 26–28]. The value of Ea for the pulmonary circulation, which can be calculated as RV ESP divided by SV, is commonly used to evaluate RV afterload.[29] The Ees/Ea ratio is a good index of ventricular-vascular coupling efficiency; the ventricle and vasculature are efficiently coupled when Ees/Ea >1, and ventricular-vascular uncoupling occurs when Ees/Ea <1.[30, 31] In the MCT-treated rats assessed here at baseline, the RV afterload Ea increased and Ees/Ea was greater than 1, suggesting that the right ventricle and pulmonary artery are efficiently coupled.

Previous studies reported that isoflurane, sevoflurane and desflurane decrease RV contractility in a dose-related manner[12, 32]. Our study showed that sevoflurane produced dose-dependent slight decreases of RV contractility (Ees, PRSW, and dP/dt_max_-EDV) in normal and PAH rats. The RV contractility, CO and EF decreased similarly after sevoflurane inhalation in both normal and PAH rats. In clinical practice, RV function in the presence of chronic PAH might be tolerant to low-concentration sevoflurane inhalation. These data indicate that sevoflurane might be suitable for sedation or as an adjunct anesthesia agent for patients with RV dysfunction.

There is growing evidence that the downregulation of SERCA2a is involved in the development of PAH and secondary RV dysfunction. The overexpression of SERCA2a has been reported to decrease pulmonary vascular remodeling, right ventricular hypertrophy and enhance cardiac contractility[33, 34]. PLB regulates the activation of SERCA2a by phosphorylation at Ser 16 and Thr 17[18]. SERCA2a/PLB interaction controls cardiac contractility, and a decrease in the SERCA2a/PLB ratio contributes to contractile dysfunction[35]. Our results showed that MCT-induced PAH is associated with downregulated SERCA2 expression, up regulation of PLB, and a decreased SERCA2/PLB ratio in the RV myocardium suggesting that RV function under the condition of PAH is potentially linked to this signal pathway. An in vitro study indicated that sevoflurane depresses the contractility of isolated myocardium by affecting SERCA2a activity and intracellular Ca^2+^ homeostasis[36]. Our results showed that sevoflurane increased PLB expression, decreased SERCA2 expression, and decreased the SERCA2/PLB ratio similarly in PAH group and NS group. Taken the RV function parameters together, these results may suggest that the impairment of the SR function causes a delay in SR calcium reuptake and consequently impairs lusitropic function, which might be associated with an impaired contractility index to PAH rats as well as the normal. The phosphorylation of PLB is known to be associated with enhanced critical left ventricular functions, such as contractility and relaxation. p-PLB/PLB ratio excluding the influence of the whole quantiity of PLB, especially in our experiment, the changing of PLB is quit obvious, is a more objective indicators compared to sole p-PLB. Interestingly, p-PLB/PLB ratio was found significantly reduced only in PAH rats after sevoflurane administration. We speculated that sevoflurane might affect the SERCA2a activities of PAH rats through dephosphorylation of phospholamban. In normal rats, p-PLB/PLB ratio was not changed indicating this dephosphorylation modulation might be unique in PAH rats compared to normal rats. At the protein level, RV remodeling might be associated with changes both the abundance and phosphorylation levels of Ca2+-handling proteins, including SERCA2a and PLB.

In conclusion, we found that sevoflurane induced a concentration-dependent depression of pulmonary, systemic hemodynamics and RV function in both normal and PAH rats. Rats with PAH can tolerate low concerntration of sevoflurane (1.5%) inhalation as well as normal rats. The SERCA2-PLB signaling pathway might play a role in the pathogenesis of PAH and effects of sevoflurane on RV function. Dephosphorylation of PLB with sevoflurance may be a unique treatment target for PAH.

## Supporting Information

S1 Fig Schematic diagram depicting the experimental protocols used in the sevoflurane inhalation experiments (TIF)

S2 Fig Midventricular transverse sections of hearts from a normal rat (A) and a PAH rat (B). The black arrows show the RV free walls. RV wall thickness increased in the PAH rat.
(TIF)
