# Supplementary figures and images for: Rats with Chronic, Stable Pulmonary Hypertension Tolerate Low Dose Sevoflurane Inhalation as Well as Normal Rats Do
Source: PLoS One. 2016 May 4;11(5):e0154154. doi: 10.1371/journal.pone.0154154 (PMC4856326; doi:10.1371/journal.pone.0154154)

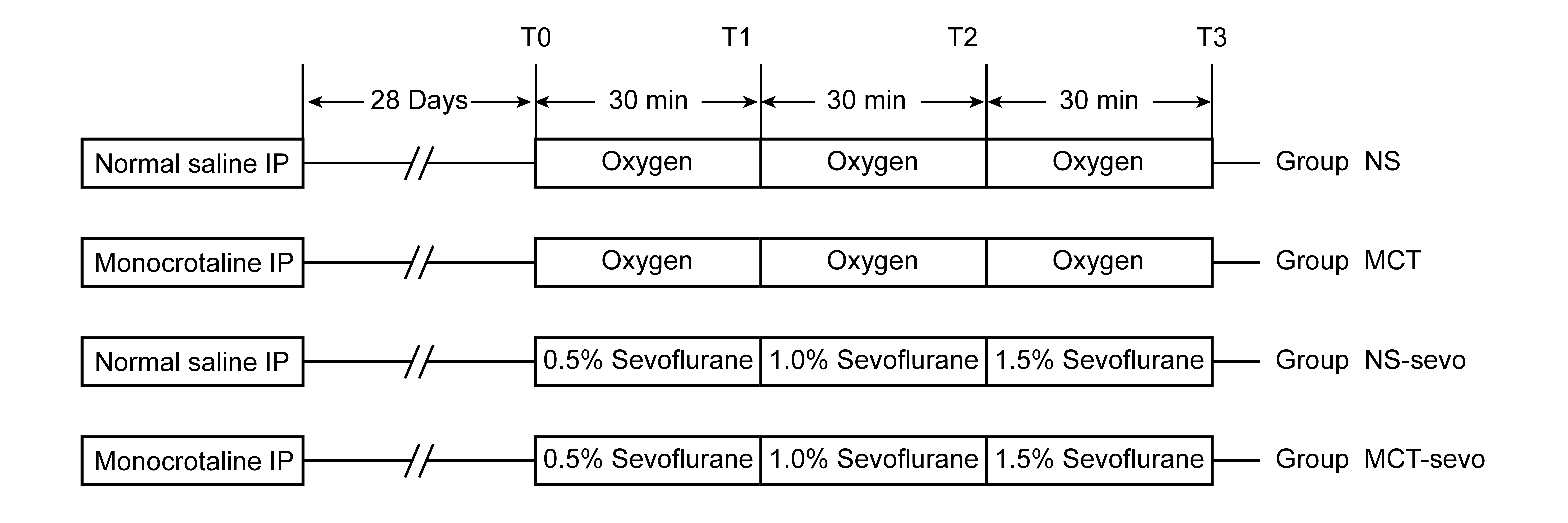

Supplement: S1 Fig — (TIF) [file pone.0154154.s001.tif]

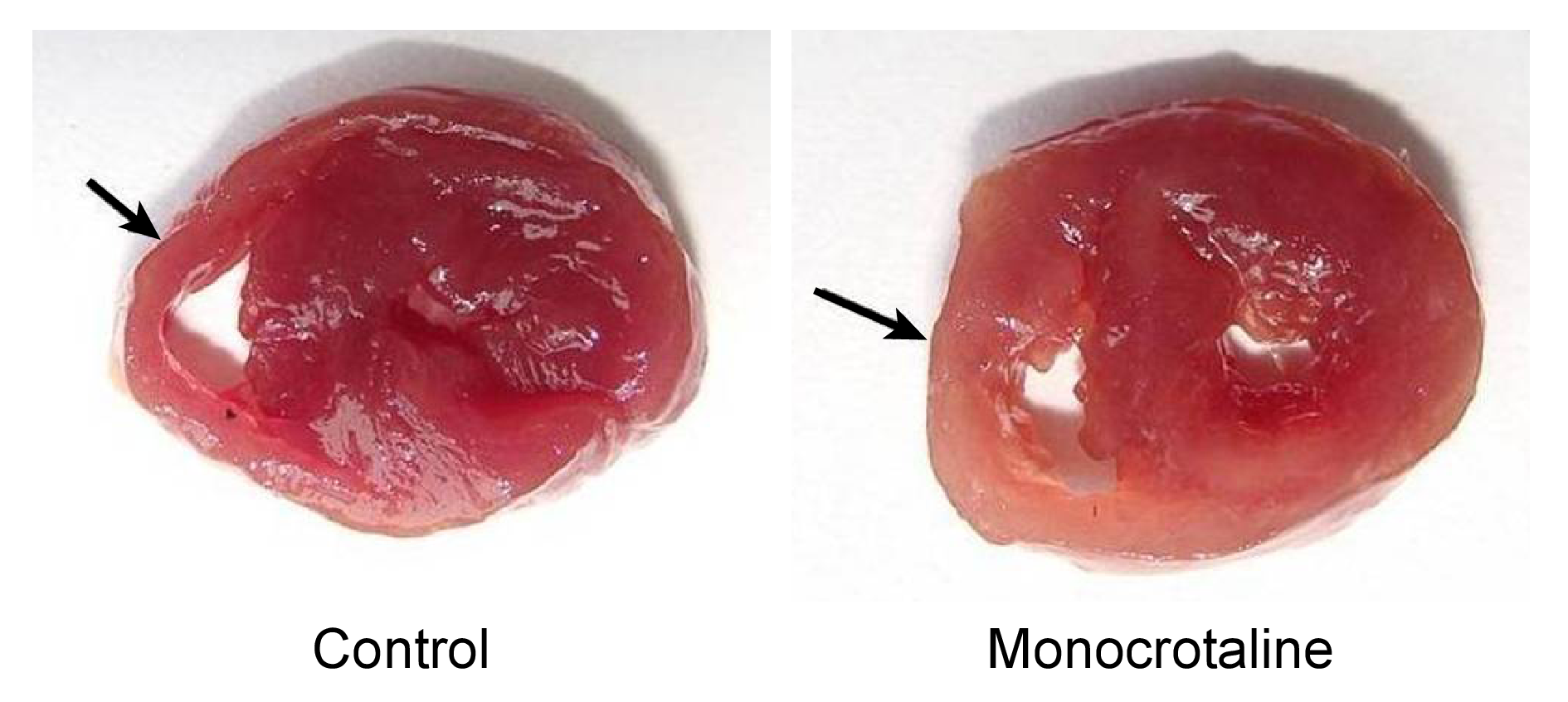

Supplement: S2 Fig — The black arrows show the RV free walls. RV wall thickness increased in the PAH rat. (TIF) [file pone.0154154.s002.tif]
